# Supplementary material for: Coupling analysis of contra-rotating fan interstage pressure pulsation and blade vibration based on wavelet reconstruction
Source: PLoS One. 2021 Feb 9;16(2):e0245985. doi: 10.1371/journal.pone.0245985 (PMC7872275; doi:10.1371/journal.pone.0245985)
Supplement: Attachment — Submitted filename: Response to Reviewers.doc [file pone.0245985.s001.doc]

Response to Reviewers

Dear Editor and Reviewers:

Many thanks for your kind help in treating our following paper with the manuscript number of PONE-D-20-29616 entitled with *Analysis of Contra-Rotating Fan Interstage Pressure Pulsation and Blade Vibration based on Wavelet Reconstruction.*

According to your instructions, we have made corresponding revisions highlighted in blue over the manuscript to clarify the points of the reviewers. I wish this revised version of manuscript could meet the standards of the reviewers and your journal and thus can be published in PLOS ONE.

The responses to the reviewers are listed below.

**Comments from Reviewer A**

1. The English writing of revised manuscript has improved a lot. However, the present form still does not meet the requirement of Plos One. Some important information is missing, such as the figures of collected signals, the sampling frequency and the signal length. In addition, the English writing of “Experimental facilities” part still need to be improved. And I suggest the authors rewrite the “Experimental methods” part. The authors should detail the experimental procedure in this part not the wavelet analysis method.

**Response:** Thanks very much for the positive comments and good suggestions from this reviewer. We have modified this manuscript under the help of American journal organizations. The English level of the manuscript has improved and the language is more fluent and readable. The English writing of **Experimental facilities** part has greatly improved than before. According to the suggestion of the reviewer, We have rewrote the **Materials and Methods** part，some unnecessary wavelet analysis methods have been removed. We also have revised the part of **Experimental methods**, some important experimental procedure information has added, such as the figures of collected signals, the sampling frequency and the Sampling time and so on. We have made corresponding revisions highlighted in blue over the manuscript to clarify the points of the reviewers.

**Comments from Reviewer B**

"In the paper “Coupling Analysis of Contra-Rotating Fan Interstage Pressure Pulsation and Blade Vibration based on Wavelet Reconstruction”, the authors Xin-Zhe Zhang and Yi-Min Li tentatively present a wavelet reconstruction analysis of flow fields from counter-rotating axial fans.

Unfortunately, the specific goals and results of the paper are poorly understood by this reviewer. In part, this is likely due to the very specific scientific nature of the manuscript (and the probably very narrow readership the paper would reach, which raises the question of whether or not it should preferably be considered in a more specialized journal). But more importantly, the manuscript does not seem yet reviewable in its current form due to its low-quality presentation.

**Response:** thank you for your professional review, we have revised the manuscript carefully, I hope it can meet your requirement. Thank you again for your comments.

The obvious limitation is the poor use of the English language. Sentences like “Meantime, there are many research use this method to do a lot of work” and many others throughout the manuscript, are indicative of a manuscript that would be in its early draft versions.

**Response:** thank you for your professional review, we have revised the manuscript carefully, especially the English language, I hope the revision can meet your requirement. Thank you again for your comments.

However, this is not the only limitation. For example, the authors refer to “J. Morlet [12], and I. Daubechies [12]”, but do not realize two flagrant errors. First, both of these references should correspond to two different papers (i.e. Morlet’s paper and Daubechies’s paper), but the authors failed to notice that the same reference number was used for both ([12]). Much more importantly, when consulting the references at the end of the document, [12] refers to another publication, by neither Morlet nor Daubechies, and from 2004. A similar example is “S. Mallat [13]”, which refers to a publication that is not by Mallat.

**Response:** thank you for your professional review, we have revised the reference carefully, and the mistake have been diminished. I hope the revision can meet your requirement. Thank you again for your comments.

Furthermore, when the authors claim “In the process of signal processing and analysis, the method of wavelet transform phase-space reconstruction [16] was applied”. The paper that is referred to here (Batchelder et al. 2014) did not present a phase-space reconstruction.

**Response:** thank you for your professional and careful review, we have revised the mistake carefully, and a new reference is added. I hope the revision can meet your requirement. Thank you again for your comments.

This indicates that the critical step a thorough proof-reading by the authors (before submitting this manuscript to a journal) has gone missing and they should not have submitted their manuscript in this state. This is especially concerning since my understanding is that this is their second resubmission. Therefore, my recommendation would be to reject the manuscript and advise the authors to consider carefully and properly re-writing it and submitting it to a more specialized journal."

**Response:** thank you for your professional and careful review, we have carried out a careful proof-reading, and the English language has been revised carefully, and many mistakes has been corrected. I hope the revision can meet your requirement. Thank you again for your comments.

Thank you very much!

Should there be any questions or suggestions, please feel free to contact me by following address,

Guojie Zhang

Zhangguojie2018@zzu.edu.cn
