# Supplementary material for: Coupling analysis of contra-rotating fan interstage pressure pulsation and blade vibration based on wavelet reconstruction
Source: PLoS One. 2021 Feb 9;16(2):e0245985. doi: 10.1371/journal.pone.0245985 (PMC7872275; doi:10.1371/journal.pone.0245985)
Supplement: Attachment — Submitted filename: Response to Reviewers.docx [file pone.0245985.s002.docx]

Dear editor and reviewers:

Thank you for your letter and the reviewers’ comments on our manuscript entitled ‘Coupling Analysis of Contra-Rotating Fan Interstage Pressure Pulsation and Blade Vibration based on Wavelet Reconstruction’ (the manuscript number is PONE-D-20-29616). Those comments are very helpful for revising and improving our paper, as well as the important guiding significance to other’s research. We have studied the comments carefully and made corrections, we hope the manuscript can be accepted for publication. The main corrections are in the manuscript and the responses to the reviewers’ comments are as follows (the replies are highlighted in red).

**Comments from the editors and reviewers:**

**Comments from the reviewers:**

**Reviewer #1:** the pressure pulsation of a contra-rotating fan is analyzed using experimental and numerical methods, including wavelet reconstruction method, which is meaningful for fan design and optimization. Although this manuscript is written well, there are some suggestions:

1. the English need be polished carefully.

**Response to the Reviewer:** Thank you for your valuable and careful comments. We have revised the paper, and the English is polished carefully. I hope it can meet your requirements. Thank you again!

2. the reference is old, some fresh references need be added.

**Response to the Reviewer:** Thank you for your valuable and careful comments. We have added lots of last references. I hope it can meet your requirements. Thank you again!

3. the numerical boundary need be explained in detailed.

**Response to the Reviewer:** Thank you for your valuable and careful comments. We have revised this section, and the detailed boundary conditions are added. I hope it can meet your requirements. Thank you again for your careful comments.

4.the conclusions need be re-written carefully to show the work’s novelty.

**Response to the Reviewer:** Thank you for your valuable and careful comments. The conclusions have been revised carefully, I hope it can show the work’s novelty and meet your requirements. Thank you again for your review again!

**Reviewer #2:** The fluid-structure coupling interaction field in the interstage of counter-rotating axial fans mainly excites the first-order vibration of the second-stage blade. The fluid-structure coupling interaction field in the interstage of counter-rotating axial fans mainly excites the first-order vibration of the second-stage blade. This research is very interesting and has some engineering application value. It is suggested to accept after modification.

1)The quality of the graph needs to be improved, whether it is compressed or not is unknown.

**Response to the Reviewer:** Thank you for your valuable and careful comments. The graph is exported by the experimental equipment. Thank you for your professional comments again!

2)The format of references is not uniform, please modify according to the standard template.

**Response to the Reviewer:** Thank you for your careful comments. We have revised the reference format following the journal requirement. Thank you again for your carefully comments again!

3)Some sentences have grammatical errors, so it is recommended to polish the professional language.

**Response to the Reviewer:** Thank you for your careful comments. We have revised the paper, and the English is polished carefully. I hope it can meet your requirements. Thank you again!
